# ERBIN limits epithelial cell plasticity *via* suppression of TGF‐β signaling

**DOI:** 10.1002/1873-3468.70121

**Published:** 2025-07-20

**Authors:** Chao Li, Gerard van der Zon, Peter ten Dijke, Tong Shen

**Affiliations:** ^1^ Oncode Institute and Department of Cell & Chemical Biology Leiden University Medical Center (LUMC) The Netherlands

**Keywords:** epidermal growth factor receptor, epithelial‐to‐mesenchymal transition, ERBIN, extracellular signal‐regulated kinase, signal transduction, transforming growth factor‐β

## Abstract

ERBIN acts as a negative regulator of the epidermal growth factor receptor (EGFR) and transforming growth factor‐β (TGF‐β)/SMAD signaling pathways that play a role in epithelial‐to‐mesenchymal transition (EMT). However, the role of ERBIN in EMT is poorly understood. Our results show that ERBIN inhibits TGF‐β‐induced EMT in NMuMG breast and in A549 lung adenocarcinoma cell lines. ERBIN inhibits TGF‐β/SMAD‐dependent gene expression and also interferes with TGF‐β‐induced extracellular signal‐regulated kinase (ERK) phosphorylation. Moreover, when the TGF‐β type I receptor kinase activity is inhibited, the mesenchymal state of ERBIN‐depleted A549 cells is reduced. Pharmacological inhibition of TGF‐β receptor and EGFR signaling counteracts increased EMT and migration in A549 ERBIN‐depleted cells. Our findings identify ERBIN as a key suppressor of EMT through coordinated inhibition of TGF‐β and EGFR signaling pathways.

## Graphical abstract

In breast and lung cancer patients, low ERBIN expression correlates with poor clinical outcomes. Here, we show that ERBIN inhibits TGF‐β‐induced epithelial‐to‐mesenchymal transition in NMuMG breast and A549 lung adenocarcinoma cell lines. ERBIN suppresses TGF‐β/SMAD signaling and reduces TGF‐β‐induced ERK phosphorylation. Pharmacological inhibition of TGF‐β receptor and EGFR signaling mitigates the enhanced EMT and migration in ERBIN‐depleted A549 cells.

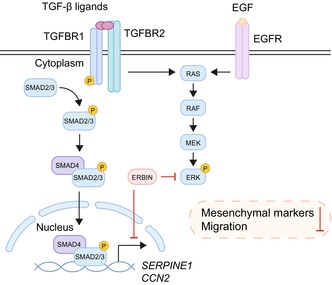

## Abbreviations

**AKT**, AKT serine/threonine kinase

**EGFR**, epidermal growth factor receptor

**EMT**, epithelial‐to‐mesenchymal transition

**ERBIN**, Erbb2/HER2‐interacting protein

**ERK**, extracellular signal‐regulated kinase

**Her2**, human epidermal growth factor receptor 2

**LAP**, Leucine‐rich repeat and PDZ domain‐containing protein

**MAPK**, mitogen‐activated protein kinase

**PDZ**, Post‐synaptic density protein 95, Discs large, Zonula occludens‐1

**Pili**, Pelitinib

**SB**, SB‐505124

**shRNA**, short hairpin RNA

**TGFBR**, transforming growth factor‐β receptor

**TGF‐β**, transforming growth factor‐β

The cytokine transforming growth factor beta (TGF‐β) plays a crucial role in regulating various cellular functions, including proliferation, differentiation, apoptosis, migration, and invasion, in a highly context‐dependent manner [1]. TGF‐β signaling occurs *via* cell surface transmembrane receptors, TGF‐β receptor type I (TGFBR1), and type II (TGFBR2), which possess intracellular domains with serine/threonine kinase activity [2]. Upon activation of TGFBR1, intracellular signaling proceeds with the phosphorylation of specific serine residues at the carboxy‐terminal end of SMAD2 and SMAD3. These phosphorylated proteins then form complexes with SMAD4, which translocate into the nucleus to modulate the transcription of target genes [3]. In addition to the canonical SMAD pathway, TGFBR1 can also initiate intracellular non‐SMAD‐mediated responses, including the activation of the extracellular signal‐regulated kinase (ERK) pathway [4, 5, 6].

Epithelial‐to‐mesenchymal transition (EMT) is a dynamic and reversible morphological process where epithelial cells lose their cell–cell contacts and apical‐basal polarity, acquiring a mesenchymal phenotype with increased cell motility. This transition is characterized by a decrease in epithelial markers, such as E‐Cadherin, and an increase in mesenchymal markers, including N‐Cadherin and Vimentin [7]. The shift from the epithelial to the mesenchymal state is often incomplete, and cells in intermediate states display mixed epithelial‐mesenchymal characteristics, a phenomenon referred to as epithelial‐mesenchymal plasticity [8]. EMT is essential during embryonic development, wound healing, and various pathological processes [9]. TGF‐β is a potent inducer of EMT, acting through TGFBR and both SMAD and non‐SMAD signaling pathways [10]. TGF‐β works in conjunction with multiple other signaling pathways to orchestrate this process [11], one of which involves the human epidermal growth factor receptor (HER2)/epidermal growth factor receptor (EGFR) signaling pathway. HER2/EGFR mediates activation of phosphatidylinositol kinase/AKT and ERK. The TGF‐β and HER2/EGFR pathways have been shown to modulate each other in various ways [12, 13, 14].

The ERBB2/HER2‐interacting protein, commonly known as ERBIN, is the founding member of the LAP (leucine‐rich repeats and PDZ) protein family. ERBIN plays a vital role in regulating cell polarity and adhesion [15, 16]. It interacts with HER2/EGFR *via* its carboxy‐terminal PDZ domain and inhibits the functions of HER/EGFR as well as other signaling pathways [17, 18]. ERBIN interacts with SMAD3 [19] and sequesters it from interacting with SMAD4, thereby inhibiting TGF‐β/SMAD3‐induced transcriptional responses [20]. ERBIN also interacts with SMAD anchor for receptor activation (SARA), which competes with SMAD2/3 for binding to ERBIN [21]. The relationship between ERBIN and epithelial cell plasticity is not yet well understood. This study provides evidence that ERBIN negatively regulates EMT by antagonizing TGFBR and EGFR signaling pathways.

## Materials and methods

### Cell culture and reagents

HEK293T (CVCL_0063), NMuMG (CVCL_0075), and A549 (CVCL_0023) cells were purchased from the American Type Culture Collection (ATCC, Manassas, VA, USA). All cell lines were cultured in Dulbecco's modified Eagle medium (DMEM; 41966029, Thermo Fisher Scientific, Waltham, MA, USA), supplemented with 10% fetal bovine serum (FBS; S1810, BioWest, Nuaillé, France) and 100 U·mL^−1^ penicillin and 100 μg·mL^−1^ streptomycin (Thermo Fisher Scientific). All mentioned cell lines were routinely maintained in a 5% CO_2_ environment at 37 °C in a humidified incubator and tested negative for mycoplasma. The authenticity of human cell lines was verified through short tandem repeats (STR) profiling. Selective small‐molecule kinase inhibitors, TβR1 type I receptor (SB505124) and Pelitinib (Peli; Tocris, Bristol, UK), were utilized at a concentration of 1 μm. Recombinant human TGF‐β3 was provided by Andrew Hinck (University of Pittsburgh, Pittsburgh, USA). The concentration of TGF‐β was applied as 2.5 ng·mL^−1^, and the same volume of ligand buffer (4 mm HCl, 0.1% (ultrapure) BSA) was used as a vehicle control. The expression vector for ERBIN [21] was provided by Carol Murphy and Theodore Fotsis (University of Ioannina, Ioannina, Greece). ERBIN was subcloned into the pLV‐puro vector.

### Lentiviral infection and generation of stable cell lines

The short hairpin RNA (shRNA) constructs for the knockdown of ERBIN were obtained from the Mission shRNA library of Sigma‐Aldrich (St. Louis, MO, USA). TRCN0000059068 and TRCN0000059070 were used for human ERBIN knockdown in A549 cells. TRCN0000313980 and TRCN0000317493 were used for mouse ERBIN knockdown in NMuMG cells.

Lentiviruses were produced by transfecting HEK293T cells with shRNA vectors targeting the mouse or human Erbin plasmid alongside three packaging plasmids: pCMV‐G (VSVG), pMDLg‐RRE (gag‐pol), and pRSV‐REV, as previously described (Zhang *et al*. 2012). The viruses were harvested 48 h post‐transfection and filtered through a 0.45 μm polyvinylidene fluoride (PVDF) membrane filter. They were either directly used for infection or stored at −80 °C to prevent loss of titer. To create NMuMG or A549 cells with ERBIN knockdown, a 1 : 1 dilution of the lentivirus in DMEM containing 5 ng·mL^−1^ Polybrene (Sigma, St. Louis, MO, USA) was prepared. Subsequently, NMuMG or A549 cells were infected with the lentiviral constructs targeting mouse or human ERBIN at a low cell density (30%). After 48 h of infection, cells were selected using 1 μg·mL^−1^ puromycin for A549 cells and 5 μg·mL^−1^ for NMuMG cells for 1 week.

### IncuCyte migration assay

Cells were seeded in IncuCyte 96‐well Essen ImageLock plates (4379; Essen BioScience, Ann Arbor, MI, USA) and scratched using the IncuCyte WoundMaker (Essen BioScience). The scratched cells were washed with phosphate‐buffered saline (PBS) and then cultured in DMEM supplemented with 1% serum within the IncuCyte live cell imaging system. Images were captured every 2–4 h over a period of 36–68 h using a 10× objective. The IncuCyte cell migration software analyzed the relative wound density for each well. All experiments were conducted with biological replicates, with representative results presented.

### Western blot analysis

Cells were washed with PBS and lysed with the lysis buffer [10% (w/v) glycerol, 2% (w/v) SDS, and 60 mm Tris–HCl (pH 6.8)] supplemented with protease inhibitor, and the cell lysates were heated at 95 °C for 5 min. SDS/polyacrylamide gel electrophoresis (PAGE) was employed to separate proteins, which were then transferred to 0.45‐μm PVDF membranes (Millipore, Burlington, MA, USA). Membranes were blocked with 5% (w/v) non‐fat milk in TBST buffer [20 mm Tris–HCl (pH 7.6), 150 mm NaCl, and 0.1% (v/v) Tween 20] for 1 h at room temperature. Primary antibodies were diluted in 3% BSA in TBST buffer and incubated with the membranes overnight at 4 °C, while secondary antibodies were diluted in 5% (w/v) non‐fat milk in TBST buffer and incubated at room temperature for 1 h. After each incubation, membranes were washed four times with TBST buffer for 5 min. Horseradish peroxidase (HRP) signals were detected using enhanced chemiluminescent (ECL) substrate (Bio‐Rad, Hercules, CA, USA) and ultra‐sensitive ECL substrate (Thermo Fisher Scientific) with a Bio‐Rad ChemiDoc imaging system. The results were subsequently processed using Image Lab software, and the antibodies used for western blot analysis are listed in Supplementary Table S1, except for anti‐p‐SMAD2 [22]. Experiments were performed at least three times, and representative experiments are shown.

### RNA extraction and quantitative real‐time PCR

Total RNA was isolated from cells using NucleoSpin RNA kits (Macherey Nagel, Düren, North Rhine‐Westphalia, Germany), according to the manufacturer's protocol. 1 μg RNA per sample was used for cDNA synthesis using RevertAid H Minus reverse transcription kit (Thermo Fisher Scientific). Quantitative PCR was performed using the GoTaq qPCR Master mix (Promega, Madison, WI, USA) on the CFX Connect Real‐Time PCR detection system (Bio‐Rad, Hercules, CA, USA). The housekeeping gene glyceraldehyde 3‐phosphate dehydrogenase (*GAPDH*) was used to calculate $2^{-\Delta \Delta C_{t}}$. The primer sequences used for RT‐qPCR are listed in (Supplementary Table S2).

### MTS cell proliferation assay

MTS assays were conducted to assess cell viability and proliferation following the manufacturer's instructions (Promega). Cells were seeded at a density of 1 × 10^3^ cells per well in 96‐well plates (Corning, Corning, NY, USA). Absorbance was measured at 490 nm using a luminometer. Each group included six biological replicates.

### Statistical analysis

All statistical analyses were performed using graphpad prism 10. Unless otherwise noted, results are presented as mean ± standard deviation (SD). Two‐sided statistical tests were utilized across all analyses, with differences considered statistically significant at *P* < 0.05.

## Results

### TGF‐β inhibits ERBIN expression

**Fig. 1:**
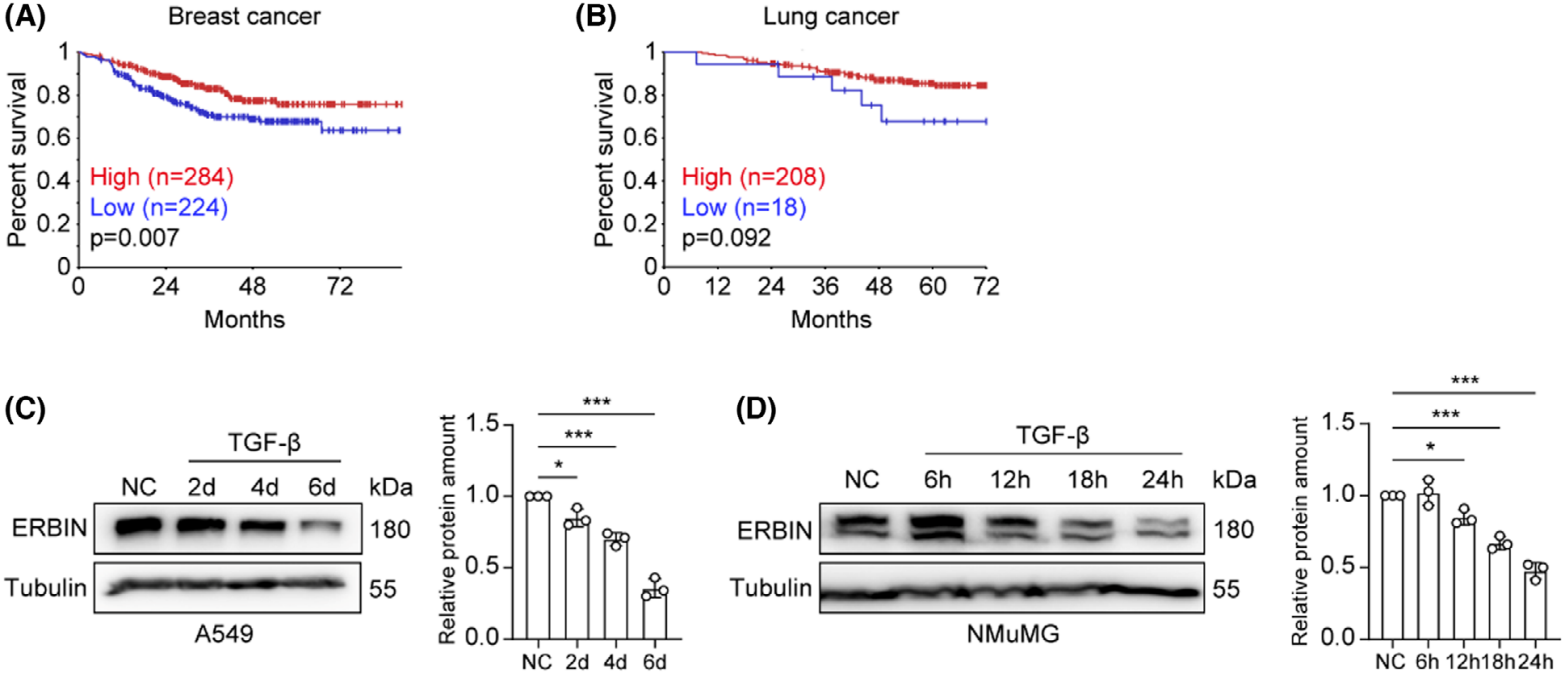
TGF‐β inhibits ERBIN expression in A549 and NMuMG cells. (A, B) Kaplan–Meier curves of breast cancer patients (GSE25066) and lung cancer patients (GSE31210) stratified by ERBIN expression. Data were generated using R2 (https://hgserver1.amc.nl/cgi‐bin/r2/main.cgi). (C, D) Western blot analysis of ERBIN in A549 and NMuMG cells treated with vehicle control or TGF‐β (2.5 ng·mL^−1^) for the indicated time, with the quantification of relative protein expression of ERBIN from *n* = 3 independent experiments. Tubulin: loading control. Means ± SD, one‐way ANOVA. *P < 0.05, ***P < 0.001.

ERBIN, initially identified as Erbb2/HER2‐interacting protein, is a negative regulator of EGFR/HER2 and TGF‐β/SMAD signaling. EGFR/HER2 and TGF‐β pathways can cooperate with each other in EMT and cancer progression. By performing Kaplan–Meier analysis of breast [23] and lung cancer patient [24] cohorts, we found that low *ERBIN* expression correlates with worse outcomes (Fig. 1A,B). To investigate a possible role of ERBIN in the interplay between these pathways and cancer progression, we examined the effect of TGF‐β on ERBIN expression in two cell types that are frequently used as models for TGF‐β‐induced EMT, that is, A549 lung adenoma carcinoma and NMuMG normal NAMRU mammary epithelial cells [25]. We found that in both cell types, TGF‐β potently inhibited ERBIN expression (Fig. 1C,D). Whereas the inhibitory effect of TGF‐β on ERBIN expression in A549 cells was more prominently observed only after 6 days of TGF‐β treatment (Fig. 1C), in NMuMG cells significant inhibition was already observed after 24 h of TGF‐β challenge (Fig. 1D).

### ERBIN inhibits TGF‐β‐induced EMT and cell migration

**Fig. 2:**
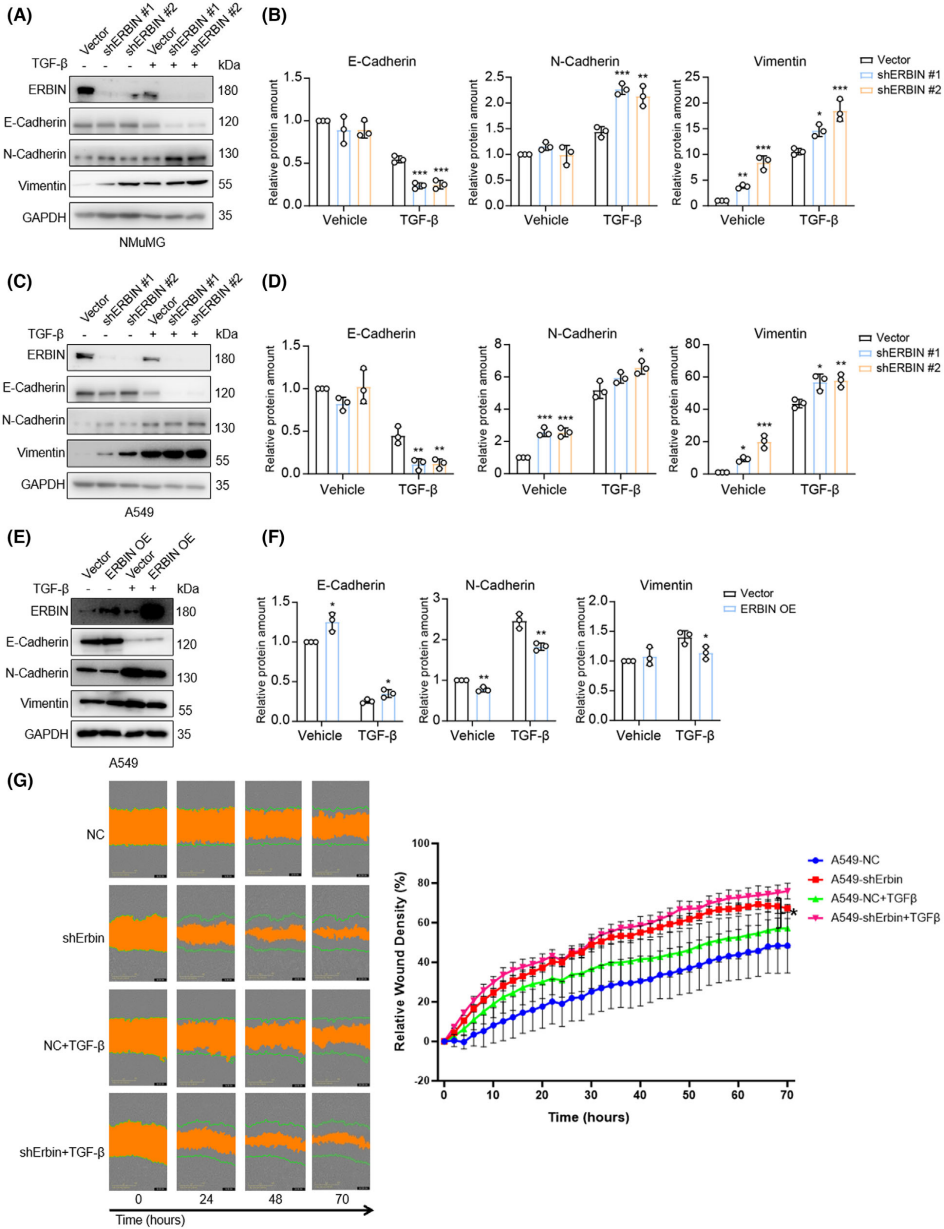
ERBIN inhibits TGF‐β‐induced EMT and cell migration. (A–D) Western blot analysis of E‐cadherin, N‐cadherin, and Vimentin expression in control or shRNA‐mediated ERBIN‐depleted NMuMG cells (A) and A549 cells (C) treated with vehicle control or TGF‐β (2.5 ng·mL^−1^) for 48 h. Results of two independent shRNAs are shown. GAPDH: loading control. The relative protein expression of E‐cadherin, N‐cadherin, and Vimentin in NMuMG cells (B) and A549 cells (D) was quantified from *n* = 3 independent experiments. Means ± SD, one‐way ANOVA. (E, F) Western blot analysis of E‐cadherin, N‐cadherin, and Vimentin expression in A549 cells either infected with control vector or ERBIN expression vector treated with vehicle control or TGF‐β (2.5 ng·mL^−1^) for 48 h. GAPDH: loading control. The relative protein expression of E‐cadherin, N‐cadherin, and Vimentin was quantified from *n* = 3 independent experiments. Means ± SD, unpaired Student's *t*‐test. (G) Analysis of TGF‐β (2.5 ng·mL^−1^)‐induced migration of control or shRNA‐mediated ERBIN‐depleted A549 cells. Representative scratch wounds are shown at the indicated time points of the experiment (left). The regions of original scratches and the areas of open area left after migrating cells are colored with green lines and orange, respectively. Relative wound density (closure) was plotted at indicated time points (right). Five biological replicates were included in this assay. Means ± SD, one‐way ANOVA. *P < 0.05, **P < 0.01, ***P < 0.001.

To examine ERBIN's effect on TGF‐β‐induced EMT, we analyzed breast [23] and lung cancer patient [24] cohorts and found that *ERBIN* positively correlates with the epithelial marker *CDH1* (encoding E‐Cadherin) and negatively correlates with the mesenchymal markers *CDH2* (encoding N‐Cadherin) and *SNAI1* (Supplementary Fig. S1A,B). Next, we depleted ERBIN in A549 and NMuMG cells and stimulated the cells with TGF‐β. Control cells were infected with a non‐targeted shRNA, and knockdown of ERBIN expression by two independent shRNAs was validated by western blotting (Fig. 2A–D). TGF‐β‐induced downregulation of E‐Cadherin and upregulation of N‐Cadherin and Vimentin was promoted upon ERBIN knockdown in both A549 and NMuMG cells. In A549 cells, also basal levels of N‐Cadherin and Vimentin (without exogenous stimulation) were increased upon shRNA‐mediated ERBIN knockdown (Fig. 2C,D). When ERBIN was ectopically expressed in A549 cells, basal E‐Cadherin levels increased and also TGF‐β‐induced downregulation of E‐Cadherin was inhibited by ectopic ERBIN expression (Fig. 2E,F). Ectopic ERBIN expression also inhibited the expression of N‐Cadherin and Vimentin (Fig. 2E,F). Together, the results suggest that ERBIN mitigates TGF‐β‐induced EMT transition. Next, we analyzed the effect of shRNA2‐mediated ERBIN depletion on the basal and TGF‐β‐induced migration of A549 cells. shRNA2 was chosen as it showed a more prominent effect than shRNA1 in EMT response. Consistent with its effect on EMT, ERBIN depletion promoted both basal and TGF‐β‐induced migration of A549 cells (Fig. 2G). Of note, depletion of ERBIN in A549 cells did not significantly alter cell proliferation (Supplementary Fig. S2).

### ERBIN inhibits TGF‐β/SMAD signaling and TGF‐β‐induced ERK phosphorylation

**Fig. 3:**
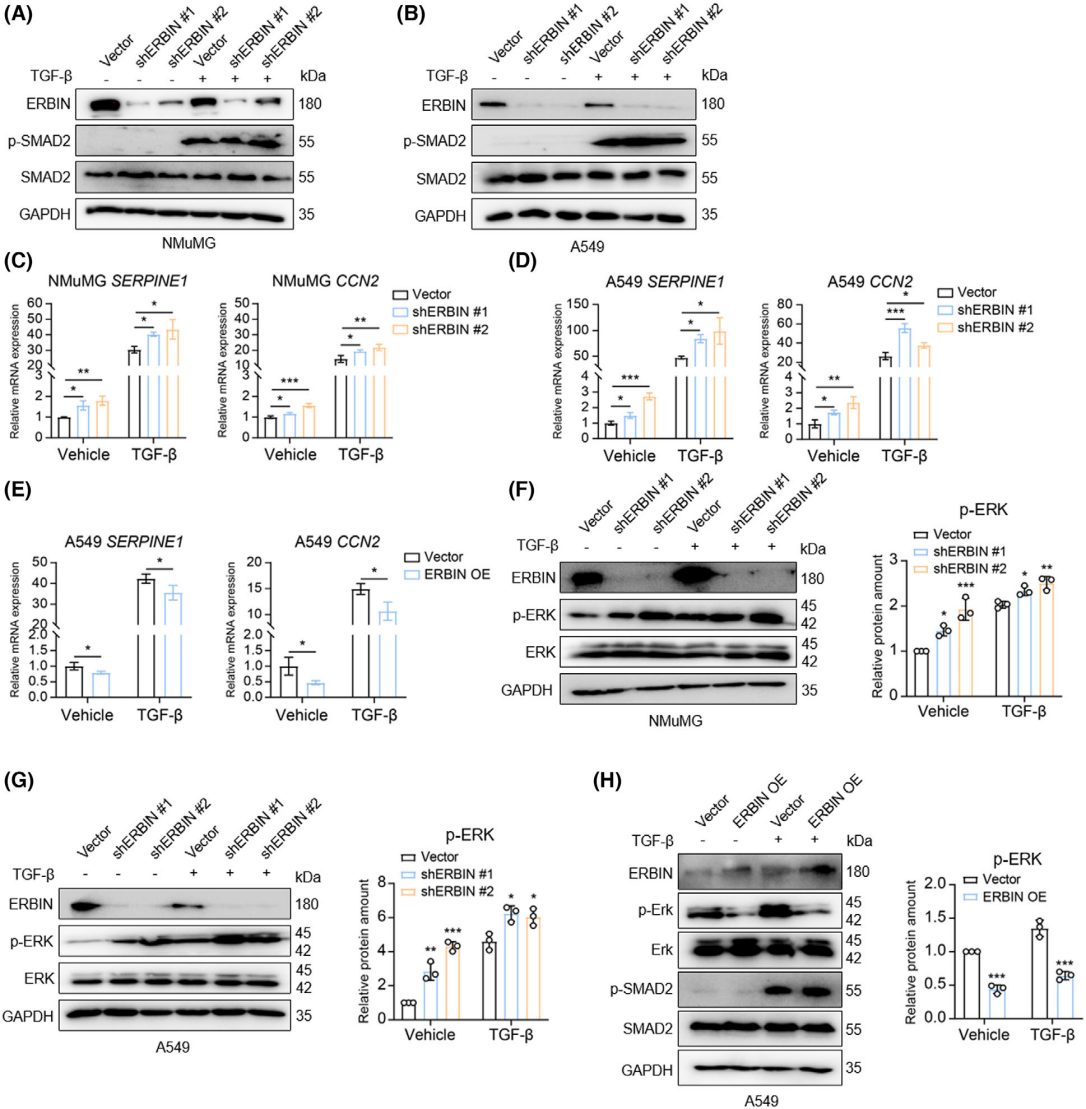
ERBIN inhibits TGF‐β‐induced ERK but not SMAD2 phosphorylation. (A, B) Western blot analysis of expression levels of ERBIN, carboxy‐terminal phosphorylated SMAD2 (p‐SMAD2), and total SMAD2 in control or shRNA‐mediated ERBIN‐depleted NMuMG (A) or A549 cells (B) treated with vehicle control or TGF‐β (2.5 ng·mL^−1^) for 1 h. (C, D) Real‐time PCR analysis of the mRNA expression of *SERPINE1* and *CCN2* in control or shRNA‐mediated ERBIN‐depleted NMuMG (C) or A549 cells (D) treated with vehicle control or TGF‐β (2.5 ng·mL^−1^) for 24 h. Means ± SD, one‐way ANOVA. (E) Real‐time PCR analysis of the mRNA expression of *SERPINE1* and *CCN2* in A549 cells either infected with control vector or ERBIN expression vector treated with vehicle control or TGF‐β (2.5 ng·mL^−1^) for 24 h. Means ± SD, unpaired Student's *t*‐test. (F, G) Western blot analysis of expression levels of ERBIN, carboxy‐terminal phosphorylated ERK (p‐ERK), and total ERK in control or shRNA‐mediated ERBIN‐depleted NMuMG (F) or A549 cells (G) treated with vehicle control or TGF‐β (2.5 ng·mL^−1^) for 1 h. Results of two independent shRNAs are shown. GAPDH: loading control. The relative protein expression of p‐ERK in NMuMG cells (F) and A549 cells (G) was quantified from *n* = 3 independent experiments. Means ± SD, one‐way ANOVA. (H) Western blot analysis of ERBIN, p‐ERK, ERK, p‐SMAD2, and SMAD2 in A549 cells either infected with control vector or ERBIN expression vector treated with vehicle control or TGF‐β (2.5 ng·mL^−1^) for 1 h. GAPDH: loading control. The relative protein expression of p‐ERK was quantified from *n* = 3 independent experiments. Means ± SD, unpaired Student's *t*‐test. *P < 0.05, **P < 0.01, ***P < 0.001.

To provide mechanistic insights into how ERBIN negatively regulates TGF‐β‐induced biological responses in A549 and NMuMG cells, we examined the effect of ERBIN depletion on TGFBR‐induced SMAD and ERK phosphorylation. We treated the cells with vehicle control or TGF‐β in complete medium for 1 h, and we observed that ERBIN depletion did not affect TGF‐β‐induced SMAD2 phosphorylation (Fig. 3A,B). We further examined the effect of ERBIN on TGF‐β signaling by measuring the mRNA levels of the downstream SMAD‐dependent target genes *SERPINE1* and *CCN2* that are activated by both SMAD and ERK signaling [26, 27, 28]. The ERBIN depletion upregulated the mRNA levels of *SERPINE1* and *CCN2* in both control and TGF‐β treatment conditions in A549 and NMuMG cells (Fig. 3C,D). The ectopic ERBIN expression downregulated the mRNA levels of *SERPINE1* and *CCN2* in A549 (Fig. 3E). In addition, we found that ERBIN depletion potentiated basal ERK and TGF‐β‐induced ERK phosphorylation in both A549 and NMuMG cells (Fig. 3F,G). Conversely, ectopic ERBIN expression inhibited both basal and TGF‐β‐induced ERK phosphorylation in A549 cells (and did not affect TGF‐β‐induced SMAD2 phosphorylation) (Fig. 3H). Taken together, Erbin can inhibit TGF‐β/SMAD signaling and TGF‐β‐induced ERK phosphorylation.

### TGFBR1 and EGFR kinase inhibition antagonize basal and TGF‐β‐induced EMT and migration in A549 ERBIN‐depleted cells

**Fig. 4:**
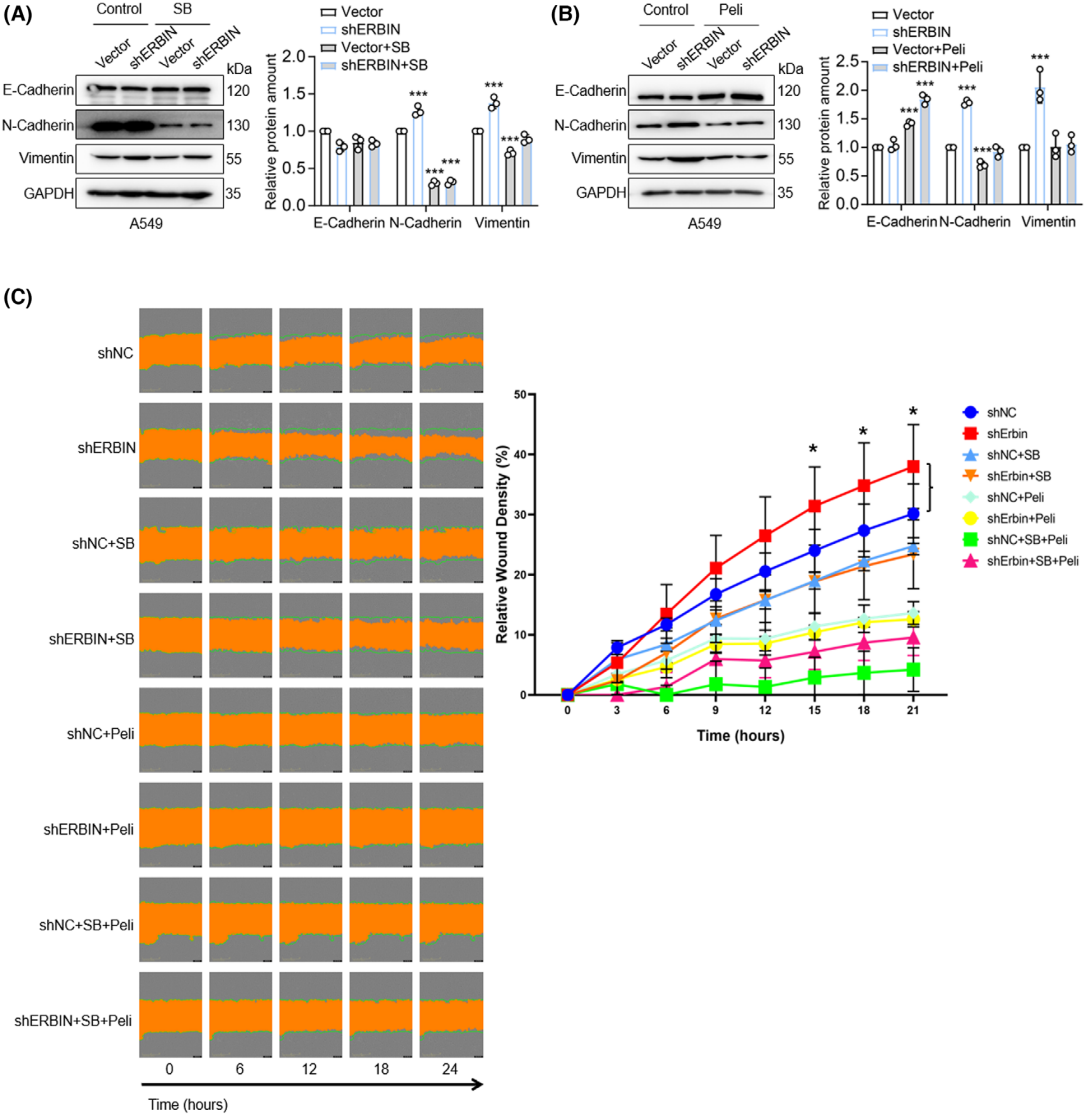
TGFBR1 and EGFR kinase inhibition antagonize basal and TGF‐β‐induced EMT and migration in A549 ERBIN‐depleted cells. (A, B) Western blot analysis of expression levels of E‐Cadherin, N‐Cadherin, and Vimentin in A549 cells upon ERBIN depletion in the absence or presence of SB (A) and Peli (B). GAPDH: loading control. The relative protein expression of E‐cadherin, N‐cadherin, and Vimentin was quantified from n = 3 independent experiments. Means ± SD, one‐way ANOVA. (C) Analysis of TGF‐β (2.5 ng·mL^−1^)‐induced migration of control or shRNA‐mediated ERBIN‐depleted A549 cells in the absence or presence of SB, Peli, or a combination of SB and Peli. Representative scratch wounds are shown at the end time point of the experiment (left). The regions of original scratches and the areas of open area left after migrating cells are colored with green lines and orange, respectively. Relative wound density (closure) was plotted at indicated time points (right). Five biological replicates were included in this assay. Means ± SD, one‐way ANOVA. *P < 0.05, ***P < 0.001.

Next, we analyzed the effect of targeting the TGFBR and EGFR kinases with SB and Peli small‐molecule kinase inhibitors on E‐Cadherin, N‐Cadherin, and Vimentin expression and cell migration in A549 vector‐transduced and ERBIN‐depleted cells. SB treatment decreased N‐Cadherin in vector‐transduced and ERBIN‐depleted cells, while the SB treatment did not affect E‐Cadherin expression (Fig. 4A). Peli treatment decreased the basal and ERBIN depletion‐induced expression of N‐Cadherin and Vimentin and increased E‐Cadherin expression level (Fig. 4B). SB or Peli treatment inhibited the A549 cell migration, and both compounds together even more effectively inhibit A549 cell migration. As A549 cell proliferation was also weakly reduced by SB and/or Peli treatment, this may also contribute to the inhibitory effect of migration observed by these compounds (Supplementary Fig. S2). ERBIN depletion in A549 cells stimulated A549 cell migration, and this effect was inhibited by SB or Peli treatment (Fig. 4C). These results indicate that targeting TGFBR1 and EGFR kinase activity can inhibit the effect of ERBIN downregulation on EMT and cell migration.

## Discussion

In this study, we show that ERBIN promotes epithelial integrity and inhibits TGF‐β‐induced EMT using A549 and NMuMG cells. Genetic manipulations of ERBIN expression were complemented by pharmacological manipulation of TGFBR and EGFR, leading to the conclusion that TGFBR and EGFR signaling pathways collaborate to promote EMT in breast and lung (cancer) epithelial cells. Moreover, consistent with this notion, our analysis of publicly available databases revealed that *ERBIN* expression inversely correlates with poor prognosis in breast and lung cancer, as well as with the expression of the mesenchymal markers *CDH2* and *SNAI1*, and positively correlates with the expression of the epithelial marker *CDH1*.

TGF‐β was found to inhibit ERBIN expression, and ERBIN was shown to inhibit TGF‐β/SMAD‐mediated gene responses and TGF‐β‐induced ERK phosphorylation in NMuMG and A549 cells. Besides TGF‐β/SMAD signaling, ERK activation has also been linked to EMT in these cells [29, 30]. Thus, the TGF‐β‐induced ERBIN downregulation may participate in a self‐enabling response toward TGF‐β‐induced EMT. Our results are consistent with a previous report that ERBIN inhibits EMT in renal tubular epithelial cells through an ERK‐dependent pathway [31].

Consistent with a previous report, we found that ERBIN did not inhibit TGF‐β‐induced SMAD2 phosphorylation [20]. However, we observed that ERBIN can negatively impact TGF‐β‐induced SMAD‐dependent transcriptional response by detecting the mRNA expression of TGF‐β signaling downstream target genes *SERPIN1* and *CNN2*. This latter effect is in agreement with a previous report [20]. ERBIN depletion promoted TGF‐β‐induced A549 cell migration. This result aligns well with the observed increase in TGF‐β‐induced EMT upon ERBIN depletion. The acquisition of cell motility is regarded as one of the core EMT changes [8]. The rescue of ERBIN depletion on A549 migration by targeting TGFBR with a small‐molecule kinase inhibitor could be obtained by the inhibitory effect on both TGFBR‐induced ERK and SMAD‐dependent responses.

Moreover, we showed that ERBIN inhibits basal TGF‐β signaling activity, ERK phosphorylation, and the mesenchymal status of cells, whereas its depletion promotes them. As cells are grown in serum, containing growth factors such as TGF‐β and EGF, this ERBIN‐induced effect may result from inhibition of TGF‐β signaling and ERK signaling mediated by TGF‐β and EGFR. Dual pharmacological targeting of TGFBR1 (using SB505124) and EGFR (using Pelitinib) antagonized most potently the TGF‐β‐induced EMT and migration in A549 ERBIN‐depleted cells. As the treatment of A549 cells with SB505124 and Pelitinib weakly inhibited cell proliferation, this may have contributed to the inhibitory effect on cell migration. Our data are consistent with the notion that dual targeting of EGFR and TGFBR may be most effective in blocking cancer cell invasion and metastasis.

Since we found that ERBIN plays a suppressive role in cancer development, pharmacologically targeting TGF‐β signaling in cancer cells may restore ERBIN expression and inhibit EMT induced by both TGF‐β stimulation and ERBIN downregulation. Our study provides further insights into the importance of ERBIN expression levels as an important determinant in TGF‐β signaling and epithelial plasticity.

## Author contributions

CL: Investigation, writing, review, and editing; GZ: Investigation, review, and editing; PD: Funding acquisition, conceptualization, project administration, writing, review, and editing; TS: Investigation, funding acquisition, writing, review, and editing.

## Conflict of interest

The authors declare no conflict of interest.

## Supporting information


**Fig. S1.** Correlation between ERBIN expression and epithelial or mesenchymal markers.
**Fig. S2.** Effect of ERBIN depletion on A549 cell proliferation.
**Table S1.** The list of antibodies for western blot.
**Table S2.** The list of primers used for real‐time PCR.

## Data Availability

The data supporting the findings of this study are available from the corresponding author upon reasonable request.
