# Supplementary material for: ERBIN limits epithelial cell plasticity via suppression of TGF‐β signaling
Source: FEBS Lett. 2025 Jul 20;599(21):3103–12. doi: 10.1002/1873-3468.70121 (PMC12599596; doi:10.1002/1873-3468.70121)
Supplement: Supplementary file 1 — Fig. S1. Correlation between ERBIN expression and epithelial or mesenchymal markers. Fig. S2. Effect of ERBIN depletion on A549 cell proliferation. Table S1. The list of antibodies for western blot. Table S2. The list of primers used for real‐time PCR. [file FEB2-599-3103-s001.docx]

Supplementary file

**ERBIN limits epithelial cell plasticity via suppression of TGF-β signaling**

Chao Li^1^, Gerard van der Zon^1^, Peter ten Dijke^1#^ and Tong Shen^1^

^1^ Oncode Institute and Department of Cell & Chemical Biology, Leiden University Medical Center (LUMC), Leiden, The Netherlands.

# correspondence:

Oncode Institute and Department of Cell & Chemical Biology, Leiden University Medical Center (LUMC), Leiden, The Netherlands.

Tel: +31 71 526 9271

Email: p.ten_dijke@lumc.nl


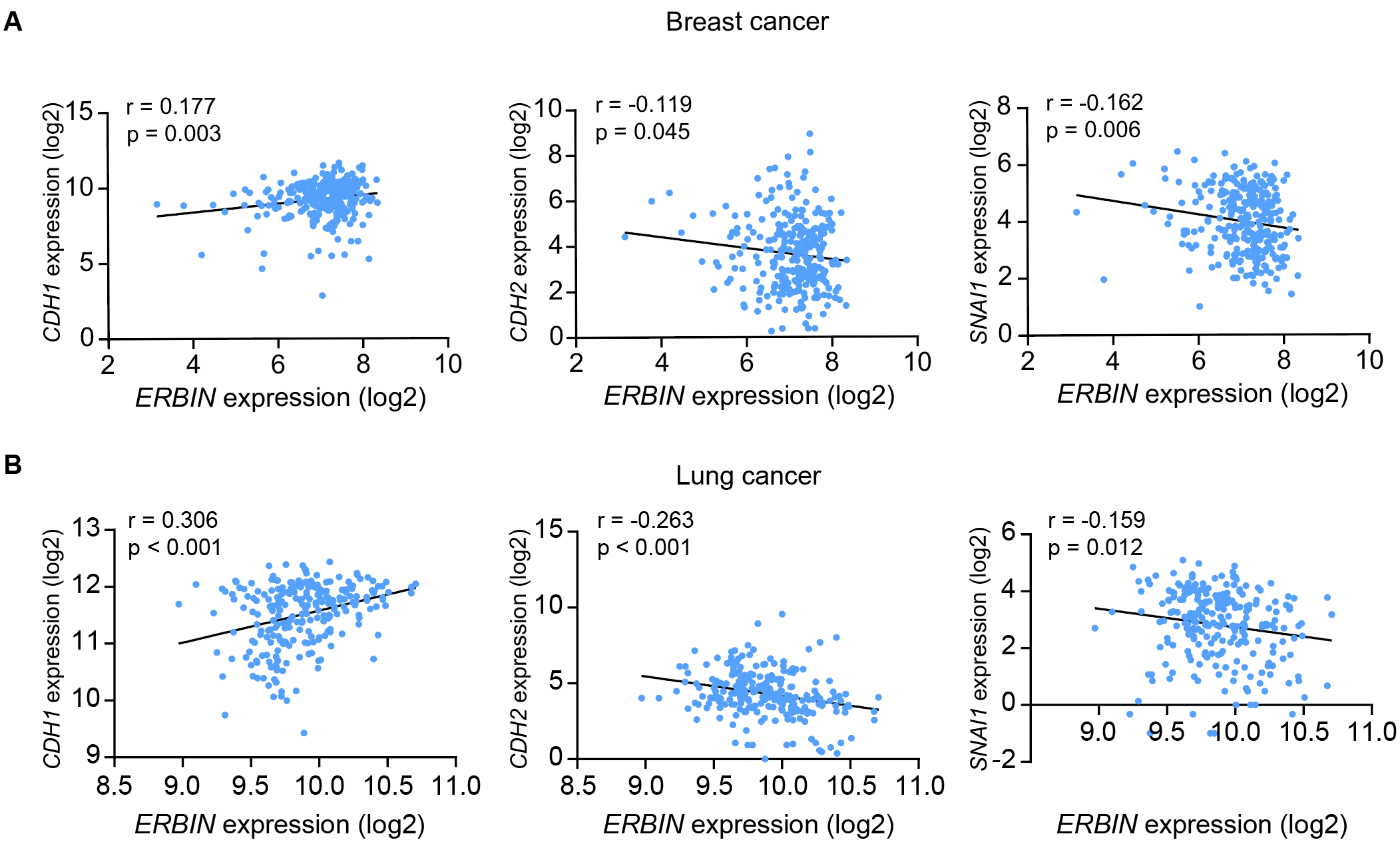


**Supplementary Fig. 1** **Correlation between ERBIN expression and epithelial or mesenchymal markers.** (A, B) Scatterplots showing the correlation between ERBIN expression and the epithelial marker (*CDH1*) or mesenchymal markers (*CDH2* and *SNAI1*) in breast (GSE25066) and lung cancer datasets (GSE31210).


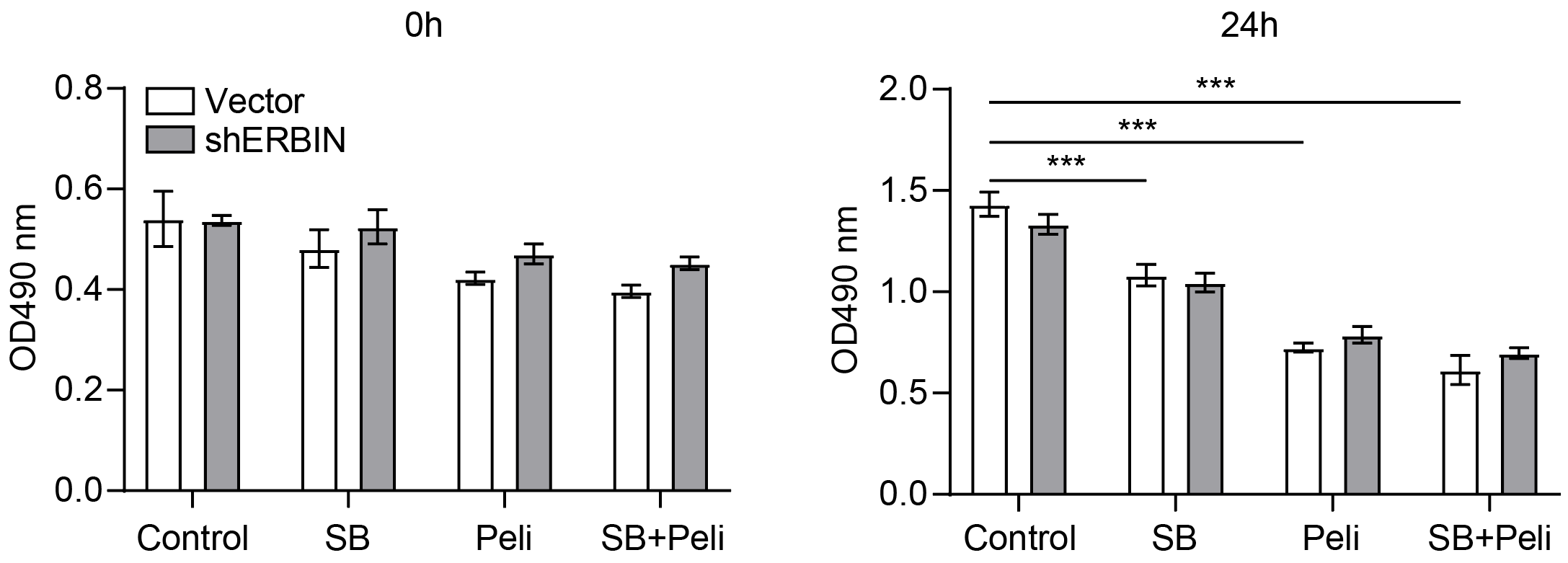


**Supplementary Fig. 2** **Effect of ERBIN depletion on A549 cell proliferation.** Effect of ERBIN depletion on A549 cell proliferation in 24 hours in the absence or presence of TGFBR1 inhibitor SB-505124 (SB, 1 μM) or EGFR inhibitor Pelitinib (Peli, 1 μM) as measured by MTS assay. Means ± SD, two-way ANOVA. *** *p* < 0.001.

**Supplementary Table 1. The list of antibodies for western blot**

| Antibody | Company | Cat # |
| --- | --- | --- |
| ERBIN | Thermo Fisher Scientific | A303-762A |
| Tubulin | Cell Signaling | 2148 |
| E-Cadherin | BD Biosciences | 610181 |
| N-Cadherin | BD Biosciences | 610920 |
| GAPDH | Millipore | MAB374 |
| Vimentin | Cell signaling | 5741 |
| SMAD2 | Epitomics | 1736-1 |
| p-ERK | Cell signaling | 9101 |
| ERK | Cell signaling | 9102s |

**Supplementary Table 2. The list of primers used for real-time PCR**

| Primer: | Target species | Sequence (5′ - 3′): |
| --- | --- | --- |
| GAPDH-F | Human | TGCACCACCAACTGCTTAGC |
| GAPDH-R | Human | GGCATGGACTGTGGTCATGAG |
| SERPIN1-F | Human | CACAAATCAGACGGCAGCACT |
| SERPIN1-R | Human | CATCGGGCGTGGTGAACTC |
| CCN2-F | Human | TTGCGAAGCTGACCTGGAAGAGAA |
| CCN2-R | Human | AGCTCGGTATGTCTTCATGCTGGT |
| GAPDH-F | Mouse | TGGCAAAGTGGAGATTGTTGCC |
| GAPDH-R | Mouse | AAGATGGTGATGGGCTTCCGG |
| SERPIN1-F | Mouse | GCCAACAAGAGCCAATCACA |
| SERPIN1-R | Mouse | AGGCAAGCAAGGGCTGAAG |
| CCN2-F | Mouse | GGCCTCTTCTGCGATTTCG |
| CCN2-R | Mouse | CCATCTTTGGCAGTGCACACT |
